# Chemical structure and genetic organization of the *E. coli* O6:K15 capsular polysaccharide

**DOI:** 10.1038/s41598-020-69476-z

**Published:** 2020-07-28

**Authors:** Hugo F. Azurmendi, Vamsee Veeramachineni, Stephen Freese, Flora Lichaa, Darón I. Freedberg, Willie F. Vann

**Affiliations:** 1 Laboratory of Bacterial Polysaccharides, Center for Biologics Evaluation and Research, Food and Drug Administration, Silver Spring, MD 20993 USA; 2 Affinivax, 650 East Kendall St, Cambridge, MA 02138 USA

**Keywords:** Biochemistry, Carbohydrates, Glycobiology

## Abstract

Capsular polysaccharides are important virulence factors in pathogenic bacteria. Characterizing the structural components and biosynthetic pathways for these polysaccharides is key to our ability to design vaccines and other preventative therapies that target encapsulated pathogens. Many gram-negative pathogens such as *Neisseria meningitidis* and *Escherichia coli* express acidic capsules. The *E. coli* K15 serotype has been identified as both an enterotoxigenic and uropathogenic pathogen. Despite its relevance as a disease-causing serotype, the associated capsular polysaccharide remains poorly characterized. We describe in this report the chemical structure of the K15 polysaccharide, based on chemical analysis and nuclear magnetic resonance (NMR) data. The repeating structure of the K15 polysaccharide consists of 4)-α-Glc*p*NAc-(1 → 5)-α-KDO*p*-(2 → partially *O*-acetylated at 3-hydroxyl of GlcNAc. We also report, the organization of the gene cluster responsible for capsule biosynthesis. We identify genes in this cluster that potentially encode an *O*-acetyltransferase, an *N*-acetylglucosamine transferase, and a KDO transferase consistent with the structure we report.

## Introduction

Capsular polysaccharides are major virulence factors of pathogenic *Escherichia coli*. These surface glycans form a coat exterior to most other surface structures such as lipopolysaccharides, protecting the bacteria from a multitude of environmental stress factors and serving as a barrier to the host immune responses^1^. The presence of specific polysaccharide capsules on the surfaces of some strains of bacteria that cause disease, make capsules, useful serological targets for monitoring and prevention of infection. *Escherichia coli* has more than 80 reported serologically distinct capsular polysaccharides, referred to as K-antigens^2^. Capsules in *E. coli* have been classified into 4 groups based on organization of the capsule synthesizing genes, mechanism of biosynthesis, and mode of transport across the membranes^3^. Groups 1 and 4 capsules use the Wzy-dependent pathway, while groups 2 and 3 make use of the ABC transporter dependent pathway of assembly and export.

Most capsules of pathogenic *E. coli* contain anionic glycan residues like hexuronic acid, *N*-acetyl neuraminic acid (NeuNAc), 2-keto-3-deoxy-D-mannooctulonic acid (KDO), and phosphodiesters^4^. Among these negatively charged components, KDO has only been identified in group 2 capsules. In capsular polysaccharide structures, KDO is usually present in a β-pyranosidic (β-KDO*p*, in *E. coli* K12, K16, K20, K23, etc.) and β-furanosidic forms (β-KDO*f*, in *E. coli* K74 and K95)^5–9^. The only known examples of KDO as α-KDO*p* in capsular polysaccharides were reported for *E. coli* K6 (LP1092) and K16^10–13^. On the other hand, α-KDO*p* is widely reported as part of the lipid A core component of the lipopolysaccharide (LPS). The genetic organization of several gene clusters encoding the biosynthesis of capsular polysaccharides containing KDO with putative glycosyltransferases have been described^14^. However, a description of the genes encoding the specific glycosyltransferases and *O*-acetyltransferase for synthesis of the K15 polysaccharides has not been reported. Therefore, determining the structure of these polysaccharides as well as the genetic organization of the biosynthetic machinery, is crucial to understanding infection and developing of effective treatments.

*E. coli* K15 is an enterotoxigenic K antigen type originally isolated from children with diarrhea and occurring at high frequency in combination with the O6 antigen and a mannose resistant hemagglutinin^15,16^. Orskov et al*.* demonstrated, in a large study of enteropathogenic *E. coli* strains from adults and children from widespread geographic locations, that some O and K serotypes frequently occurred together^17,18^. They suggested that these strains represent clones which have adapted to growth in the small intestine. A structure was reported for the *E. coli* K15 polysaccharide without experimental details of how this structure was determined nor the source the polysaccharide^7^. Significantly, this reported structure differs from our findings. In this study, we describe structural analysis of capsular polysaccharide isolated from *E. coli* str. F8316/41 (O6:K15:H16). We also report the partial characterization of the gene cluster responsible for biosynthesis of the K15 capsular polysaccharide in the same strain of *E. coli* K15. This gene cluster is very similar to that of *E. coli* K15 strain 536^19^ whose polysaccharide structure has not been reported.

## Results and discussion

### Composition of the K15 polysaccharide

**Figure 1:**
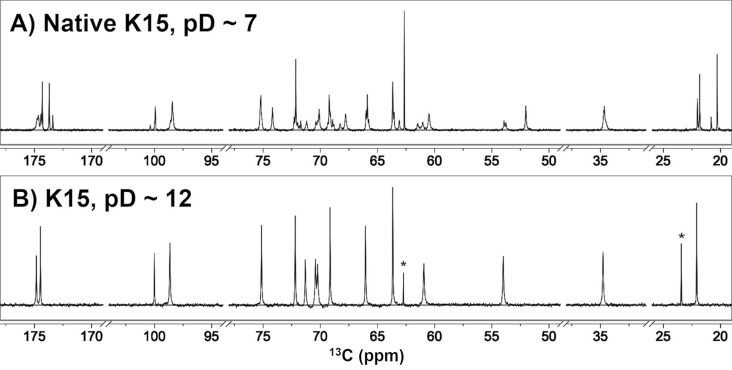
^13^C 1D NMR of the K15 polysaccharide at 50 °C. (**A**) Native K15 at pD ~ 7. (**B**) K15 PS after raising the pD to ~ 12. A total of 16 signals can be identified belonging to the K15 PS in panel (**B**); the ‘*’ marks indicate narrow trace signals from K15 decomposition (acetate and glycerol).

The composition of the K15 capsular polysaccharide was determined by a combination of chemical degradation methods and analysis of its one-dimensional carbon nuclear magnetic resonance (1D ^13^C-NMR) spectrum. The polysaccharide is composed of two monosaccharides, *N*-acetylglucosamine (GlcNAc) and KDO. *N*-acetylglucosamine and KDO were detected exclusively in acid methanolysates by gas-chromatography mass-spectrometry (GC–MS) as trimethylsilyl (TMS) derivatives. The identity of the hexosamine in the polysaccharide was confirmed by ninhydrin degradation of an acid hydrolysate. The product of the ninhydrin degradation was identified chromatographically as arabinose, the expected degradation product for glucosamine^20^. KDO was identified by acid hydrolysis of the carboxyl reduced polysaccharide, conversion to alditol acetates and analysis by GC–MS for characteristic fragmentation patterns. These composition results are in agreement with the chemical shift location and number of carbon signals observed in 1-D ^13^C -NMR spectra (Fig. 1). Treatment of the native polysaccharide (Fig. 1A) with dilute sodium hydroxide results in loss of methyl and carbonyl carbon signals, simplifying the spectrum (Fig. 1B). The alkali treated polysaccharide has two chemical shifts in the anomeric region at 98.5 and 100 ppm, two carbonyl chemical shifts at 174.46 and 174.83 ppm and a single methyl chemical shift at 22.09 ppm. The loss of some methyl and carbonyl carbon signals under alkaline conditions with a corresponding simplification of the spectrum is characteristic of an *O*-acetylated polysaccharide^21^.

The presence of only two resonances of similar intensities in the anomeric region of the ^13^C -NMR spectra of the de-*O*-acetylated polysaccharide suggests a disaccharide repeat unit. The reduction of the number of signals after de-*O*-acetylation to 16 and their chemical shift positions are expected for a disaccharide repeat unit composed of an *N*-acetylhexosamine and a deoxy acidic sugar with eight carbons, i.e., KDO.

An oligosaccharide consisting of KDO and GlcNAc was isolated by selective cleavage at the acid labile KDO linkage with 1% acetic acid. This oligosaccharide eluted on a BioGel P2 gel filtration as a disaccharide, which is in agreement with the suggestion of a disaccharide repeat unit from the NMR evidence above. Methylation analysis of the purified oligosaccharide yielded only 3,4,6-*O*-methyl-*N*-acetylglucosamine, as expected for a terminal GlcNAc, indicating that the repeat unit consists of a disaccharide of GlcNAc and KDO.

### Substitution of repeat unit

**Figure 2:**
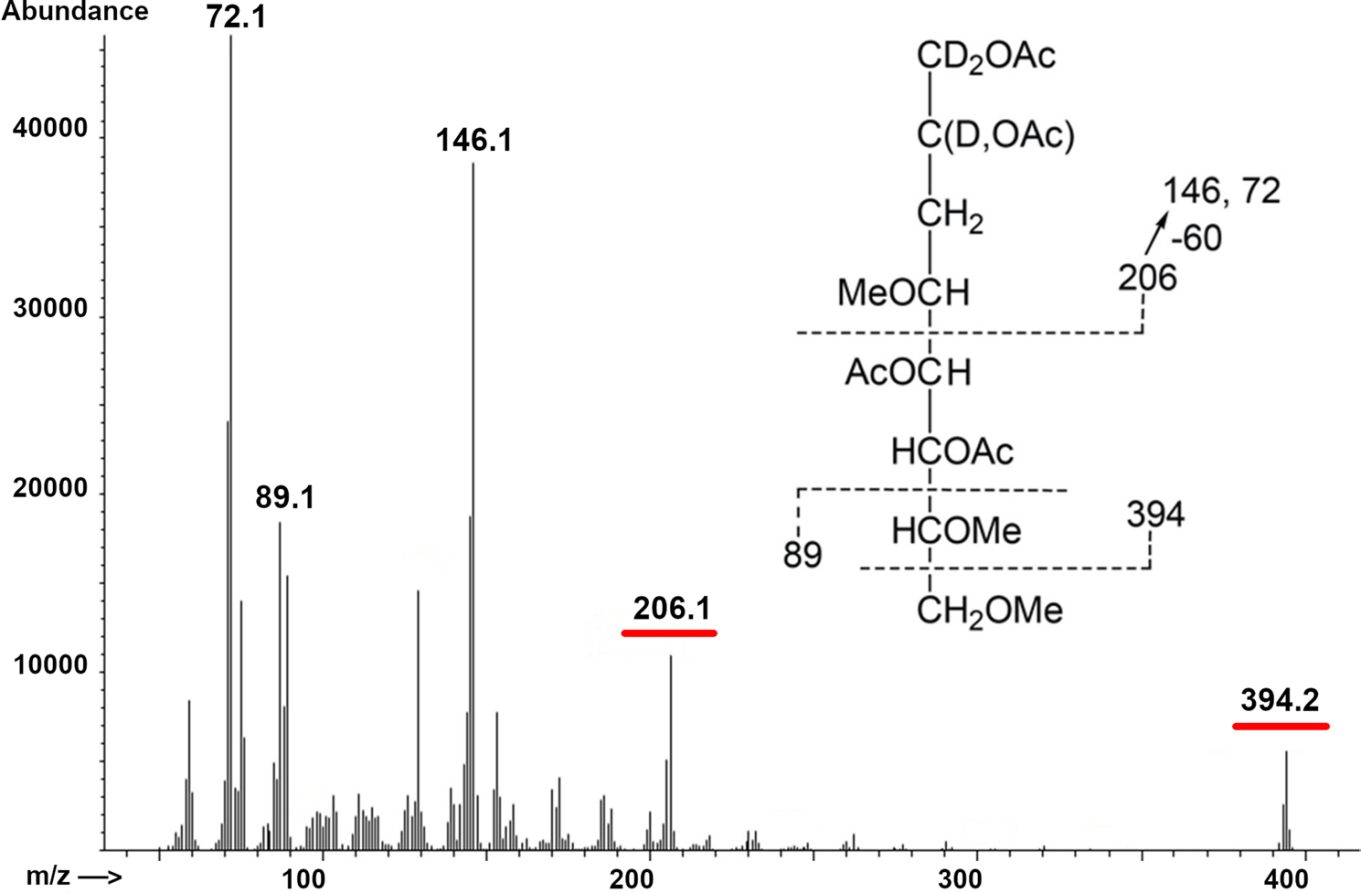
Mass spectrum and fragmentation profile of 2,5 linked KDO labeled peak identified in reduced permethylated K15 polysaccharide. Carboxylate reduced permethylated K15 polysaccharide was hydrolyzed, converted to alditol acetates and analyzed by GC–MS.

The substitution pattern of the monosaccharides in the polysaccharide repeat unit was determined by two dissimilar methylation analysis experiments, due to the difference in lability of GlcNAc and KDO. The GlcNAc linkage was determined by permethylation of the polysaccharide and subsequent analysis by GC–MS of the resulting alditol acetate, which yielded only 3,6-*O*-methyl-*N*-acetylglucosamine, suggesting that this glycan is 4-substituted. A permethylated KDO residue was not conveniently detected during this analysis, thus another approach was taken. The permethylated K15 was treated with LiBD_4_ to reduce the carboxylate group, and subsequently hydrolyzed with TFA and converted to alditol acetates before GC–MS. The major fraction detected in this analysis corresponded to a 2,5 linked KDO suggesting that the KDO is 5-substituted (Fig. 2). This was further confirmed by the alkali lability of the periodate oxidized polysaccharide. The K15 polysaccharide is oxidized by periodate with concomitant loss of KDO but remains polymeric. The oxidized product is depolymerized by mild alkali, suggesting a β-elimination reaction. Depolymerization was easily followed by TLC, which showed the disappearance of immobile polysaccharide and the appearance of a component with R_fGlcNAc_ = 0.75. Thus, the chemical analysis indicates that the repeat unit of the K15 CPS is a disaccharide of 4-substituted GlcNAc and 5-substituted KDO.

**Figure 3:**
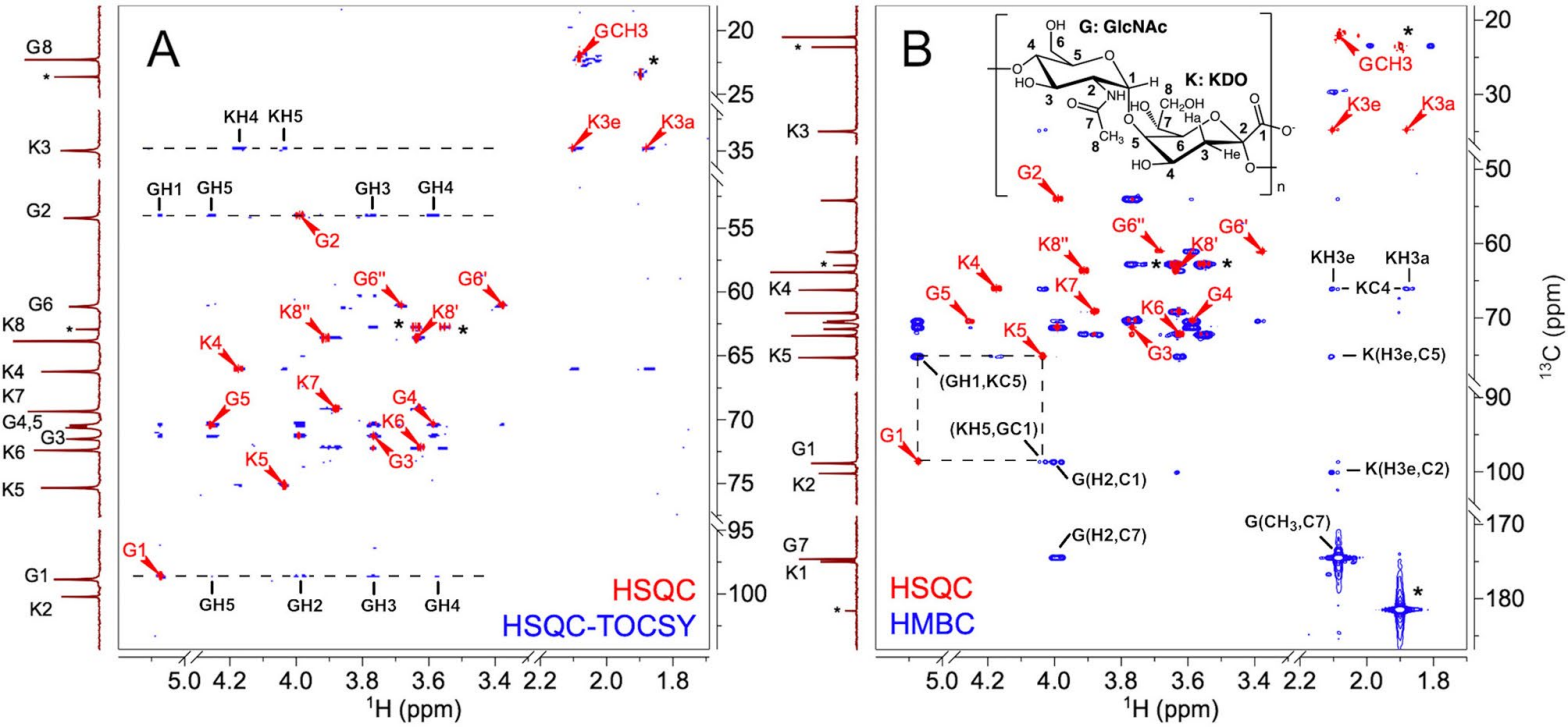
NMR assignments of the K15 CPS at pD ~ 12 and T = 50 °C. For reference the same HSQC is overlaid in both panels (red) with the C-H peak assignments, and a ^13^C 1D spectrum is shown as vertical trace. (**A**) HSQC-TOCSY spectrum acquired with a 60 ms mixing time (blue), showing the ^1^H–^1^H spin coupling at different ^13^C chemical shifts. Horizontal dashed lines highlight examples of ^1^H–^1^H spin systems: at GlcNAc C1 (98.55 ppm, indicated by G1), GlcNAc C2 (53.97 ppm, indicated by G2) and KDO C3 (34.75 ppm, indicated by K3) ^13^C chemical shifts. (**B**) HMBC spectrum (blue) for long-range connectivity was assigned by NMR. The G1-K5 HMBC interglycosidic correlation confirms the linkage in the repeating disaccharide and is indicated by dashed lines. The ‘*’ marks indicate trace signals from K15 decomposition (acetate and glycerol). (A high-resolution image of panel A is provided as Figure S1).

The analysis above was confirmed by NMR experiments. We used the following process to confirm the composition and substitution of the repeat unit. The HSQC-TOCSY^22,23^ experiment produces signals from single-bonded CHs correlated to ^1^H signals from Hs belonging to the same ring as the CH. Since there are multiple carbon atoms in each ring, this experiment yields redundant information that allows us to confirm ^1^Hs assignments. An overlay of the ^1^H–^13^C HSQC and HSQC-TOCSY (Fig. 3A), allows assignment of the ^1^H and ^13^C chemical shifts in the KDO and GlcNAc rings, as follows. In the HSQC-TOCSY, cross-peak intensities originating from a given ^1^H decrease as the number of bonds between the ^1^Hs increases, thus suggesting tentative assignments based on relative intensities. For instance, all ^1^H resonances in the GlcNAc ring can be identified and assigned tentatively from the ^13^C slice corresponding to G-C1 (Fig. 3A). The anomeric G1 is found at ~ (98.6, 5.07) ppm in ^13^C and ^1^H, respectively (Table 1). From G1 it is possible to use the HSQC-TOCSY to provisionally assign all ^1^H resonances through G5. Signals from H5-C5 and H4-C4 have lower intensity than those from H3-C3 and H2-C2. Similarly, the characteristic signals for the equatorial and axial H3′s in KDO, H3e,a, respectively in KDO^24,25^, allow tentative assignment of H4 and H5 at the C3 slice (~ 34.7 ppm).

**Table 1:** ^1^H and ^13^C NMR assignments for K15 ± (OAc).

Bond	K15(-OAc)		K15(G3 + OAc)	
	δ_C_ (ppm)	δ_H_ (ppm)	δ_C_ (ppm)	δ_H_ (ppm)
G1	98.55	5.070	98.46	5.127
G2	53.97	3.988	52.05	4.219
G3	71.25	3.769	74.20	5.132
G4	70.25	3.589	67.84	3.796
G5	70.41	4.257	70.14	4.369
G6'	61.1	3.387	60.5	3.400
G6′'	61.1	3.692	60.5	3.708
CH_3_(NAc)	22.09	2.083	21.88	2.027
C(NAc)	174.46	–	174.36	–
CH_3_(OAc)	–	–	20.34	2.103
CO(OAc)	–	–	173.76	–
K1	174.83	–	174.81	–
K2	100.01	–	99.95	–
K3e	34.75	1.887	34.67	1.946
K3a	34.75	2.098	34.67	2.125
K4	65.92	4.167	66.02	4.187
K5	75.1	4.037	75.25	4.079
K6	72.18	3.612	72.30	3.592
K7	69.2	3.891	69.2	3.891
K8'	63.6	3.636	63.7	3.576
K8′'	63.6	3.915	63.7	3.923

To confirm the initial assignments and complete the remaining ones, we added to our analysis a low-pass filtered HMBC^26,27^, which produces only long-range ^1^H–^13^C correlations. Figure 3B shows an overlay of the HSQC (red) with the HMBC (blue) spectra. The presence of peaks depends on both, the ^1^H–^13^C couplings between each specific pair of atoms and multiple nuclear relaxation rates. Consequently, not all potential signals are observed. For instance, there is no cross-peak for (GH1- GC2) but there is one between a related pair of nuclei, GH2 and GC1. The HMBC also provided evidence of the G-K linkage: a cross-peak between G1 and K5, indicated a G1-K5 linkage between the glycan residues. In addition, the ^13^C chemical shift assignments of K2 (δ_C_ = 100.0 ppm) and *N*-acetyl carbonyl group (δ_C_ = 174.5 ppm) are confirmed through the cross-peaks K(H3e, C2) and G(CH3, C7), respectively. This indicates that the other carbonyl peak (δ_C_ = 174.8 ppm) corresponds to K1.

**Figure 4:**
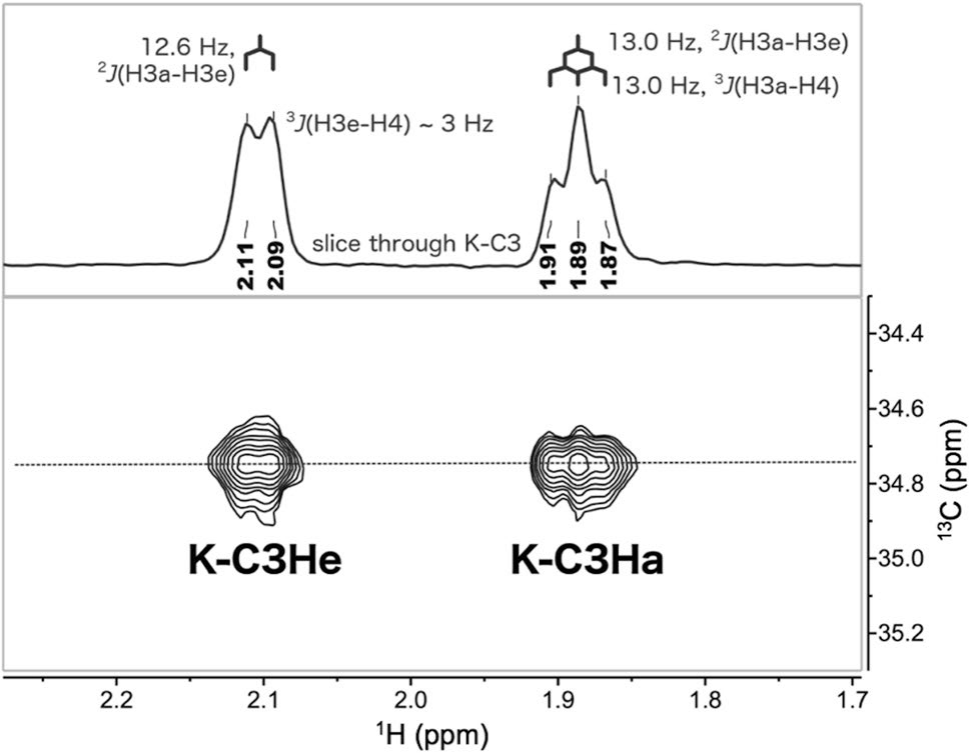
High resolution ^1^H–^13^C HSQC of the K15 PS at 70 °C, showing the couplings to K-H4 from K-H3a and K-H3e. The two different ^3^*J*_HH_ couplings to K-H4 in KDO allowed the assignment δH at 2.10 ppm as the equatorial H3 (K-H3e) and the δH at 1.89 ppm as the axial H3 (K-H3a).

The analysis continued with the ^1^H assignments of the H3a and H3e of KDO in the K15 polysaccharide using a ^1^H–^13^C high-resolution HSQC at 70 °C, which allowed determination of ^1^H–^1^H couplings between K-H3a/e and K-H4 (Fig. 4). H4 is axial in KDO*p*, the resulting ^3^*J*_*H3aH4*_ is large (H4-C4-C3-H3a torsion of ~ 174°) and similar to ^2^*J*_*H3aH3e*_ in magnitude, while ^3^*J*_*H3eH4*_ is small (H4-C4-C3-H3e torsion of ~ 56°) compared to ^2^*J*_*H3aH3e*_. Thus, K-H3e displays a doublet at 2.10 ppm (^3^*J*_HH_ = 12.6 Hz) while K-H3a results in a triplet at 1.89 ppm (^3^*J*_HH_ = 13.0 Hz).

**Figure 5:**
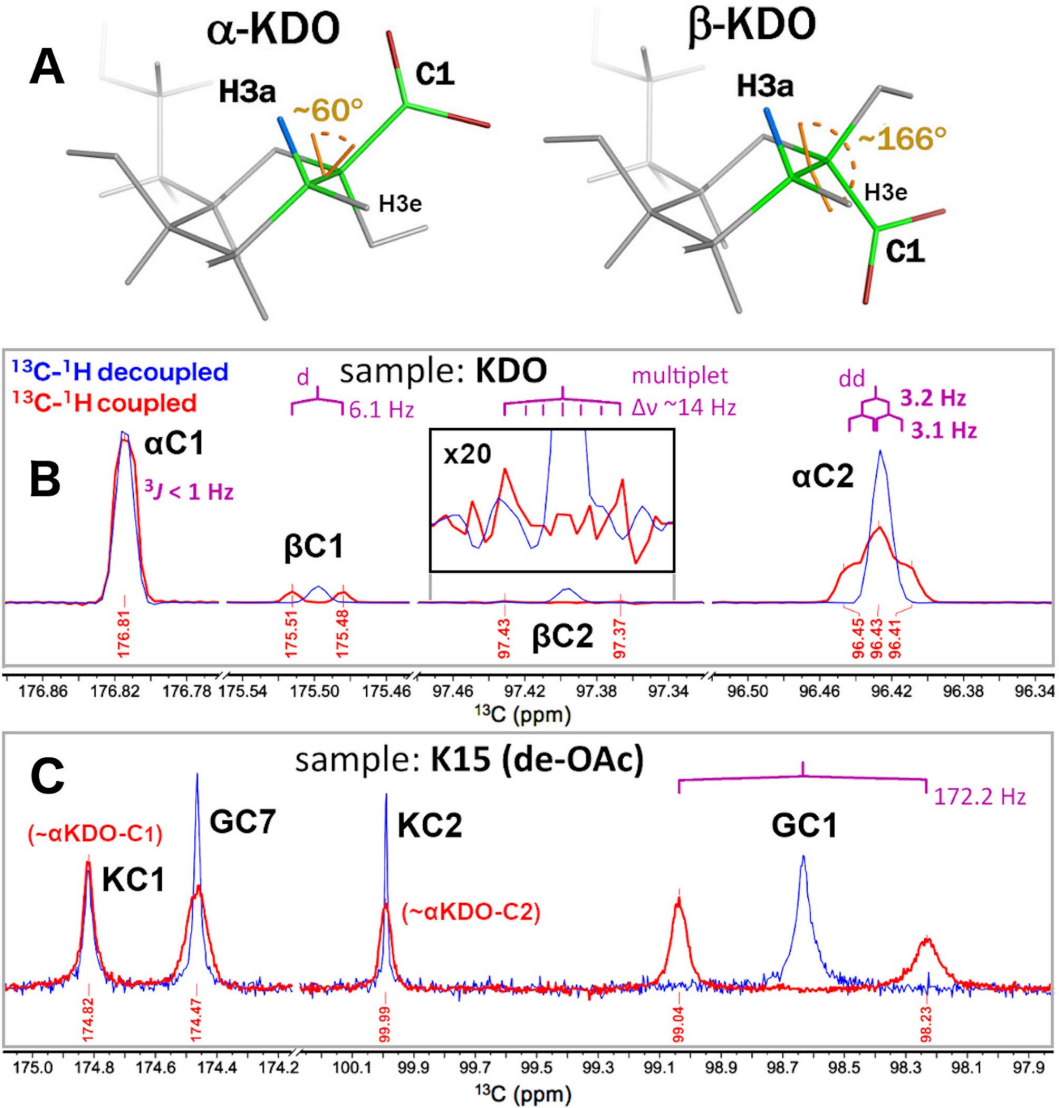
Comparison of the ^13^C (^1^H coupled and decoupled) α- and β-anomers of KDO monomer signals, to those of the K15 capsular polysaccharide signals for determination of the anomeric configuration of KDO in K15. (**A**) Schemes showing the H3a to C1 three-bond torsion in α- and β-KDO, respectively. (**B**, **C**) ^13^C ^1^H -coupled (red) and ^13^C ^1^H -decoupled spectra of KDO (**B**) and K15 (**C**), demonstrating that α-KDO is the K15 constituent. Figure 5A was created with PyMol 2.4 (www.schrodinger.com/pymol); the coordinates for α/β-KDO were obtained from CarbBuilder (www.organ.su.se/gw/doku.php?id=carbbuilder).

Multiple NMR parameters are indicative of the anomeric configuration of pyranose KDO (KDO*p*, Fig. 5A), most notably the δ_H_ of the axial and equatorial H3 (H3a and H3e, respectively)^28^, and the C-H three-bond coupling (^3^*J*_CH_) between H3a and C1^29^. From δ_H_ analysis, it has been observed that a reliable criterion for distinguishing αKDO and βKDO is the signal separation between δH3a and δH3e (or Δ(δH3a, δH3e))^13^; for αKDO Δ(δH3a, δH3e) < 0.4 ppm, while for βKDO Δ(δH3a, δH3e) > 0.4 ppm (Supplementary Table S1). For K15 Δ(δH3a, δH3e) = 0.21 ppm, further validating that KDO is in the α-configuration in K15. Based on a representative survey (Supplementary Table S1), we propose that the same conclusion can be reached using only the δH3e, as for all reported values it is verified that δH3e(αKDO) < 2.3 ppm and δH3e(βKDO) > 2.3 ppm. For K15 the δH3e signal is ~ 2.1 ppm, well below the 2.3 ppm threshold, further confirming the configuration as αKDO.

The ^3^*J*_CH_ criteria is based on the fact that the H3a-C3-C2-C1 torsion for the β-configuration is ~ 166°, which results in a ^3^*J*_CH_ coupling of ~ 6 Hz. For the α-configuration the same torsion is ~ 60°, yielding a K(H3a-C1) ^3^*J*_CH_ coupling of < 1 Hz (Fig. 5B). The torsion between H3e and C1 is about the same (~ 60°) for both α- and β-KDO, thus, the ^3^*J*_CH_ value for the H3e-C3-C2-C1 torsion is not informative for establishing the α or β anomeric configuration in KDO. For the K15 PS, the KC1 (174.8 ppm) and KC2 (100.0 ppm) ^13^C 1D signals (Fig. 5C) are broad preventing a precise measurement of the couplings, however, these peaks are similar to those in Fig. 5B arising from αKDO-C1 (176.8 ppm) and C2 (96.4 ppm), and dissimilar to those arising from βKDO-C1 (175.5 ppm) and C2 (97.4 ppm) signals, providing independent confirmation of the presence of α-KDO in the K15 PS.

The δ_H_ of ~ 5.1 ppm indicates that GlcNAc is in the α-configuration, as δ_H_ ranges between 4.4 and 4.8 ppm for β-GlcNAc-H1^30,31^. The measured one-bond coupling (^1^*J*_CH_) for GlcNAc-C1H1 is ~ 172 Hz (Fig. 5C), also indicating that the GlcNAc ring is in the α configuration, expected ^1^*J*_CH_ values for β-GlcNAc-C1H1 are about 161 Hz^32^.

The native (*O*-acetylated) PS shows several NMR signals that cannot be directly identified by comparison between the native and de-*O*-acetylated NMR spectra (Supplementary Fig. S2A). Signals for the native PS, were completely assigned running the same set of experiments as for the de-*O*-acetylated sample and indicated that G-C3 is *O*-acetylated. *O*-acetylation deshields the G-H3 signal by ~ 1.35 ppm, by far the most pronounced change in δ_H_ for K15 resulting from *O*-acetylation. The δ_H_ at ~ 5.1 ppm of G-H3-OAc is typical for *O*-acetylated positions^33^.

Table 1 contains the ^1^H and ^13^C NMR assignments for de-*O*-acetylated K15, and the major population of native K15. Except for the *O*-acetylated G3 and its immediate neighbors, few chemical shifts differ for the native and de-*O*-acetylated forms.

**Scheme 1:**
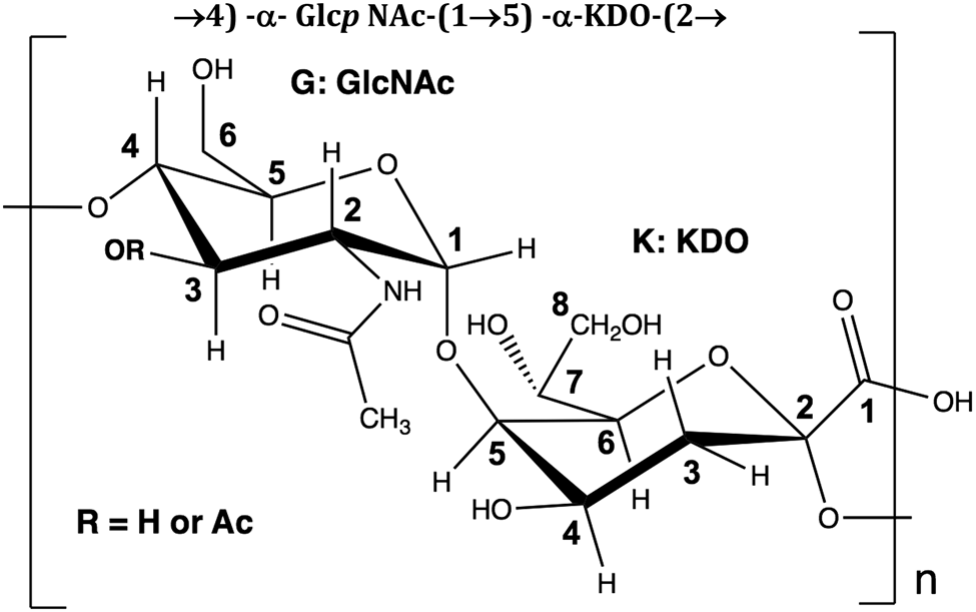
Chemical structure of the disaccharide repeat unit of the K15 polysaccharide.

To obtain an approximate *O*-acetylation ratio in the K15 CPS, we ran a long 1D inverse-gated ^13^C experiment to quantitate, by integration, isolated NMR signals of the *O*-acetylated PS and compare it with signals comprising total PS. Supplementary Fig. S2B shows this comparison, illustrating that ⪞ 60% of GlcNAc residues are *O*-acetylated at the C3 position. Altogether, the data indicate that the K15 repeating unit consists of the structure in Scheme 1.

### Sequencing the *E. coli* strain F8316/41(O6:K15:H16) and capsule gene cluster identification

The genetic organization of the capsular gene cluster (in pathogenic island V) of *E. coli* strain 536 (O6:K15:H31) was previously reported to be a combination of structural features from group 2 and 3 capsule determinants^19^. However, the lack of insight into the capsule specific genes in region 2 and the unusual organization of the strain 536 gene cluster, prompted us to study the capsular gene cluster from *E. coli* strain F8316/41 (O6:K15:H16), from which the capsular polysaccharide was isolated for determining the structure. The chromosomal DNA isolated from *E. coli* strain F8316/41 was sequenced using single-molecule real-time (SMRT) sequencing technology^34^ yielding five contigs (GenBank accession numbers: JAADZB010000001-JAADZB010000005). The annotated DNA contigs were screened for gene markers reported to exist in proximity to Group 2 and Group 3 capsular gene clusters of *E. coli* that use the ABC transporter pathway, namely *pgk* (gene encoding phosphoglycerate kinase), *pheV* (gene encoding phenylalanine tRNA)and *serA* (gene encoding phosphoglycerate dehydrogenase)^35–37^. All these gene markers along with a second 22 bp truncated copy of *pheV* (*pheV’)* were identified in the second contig (GenBank accession numbers are provided in Supplementary Table S2). We also observed that 20 kb of DNA downstream to *pheV’,* contained open reading frames (ORF’s) that closely resembled K15 capsular gene cluster from *E. coli* 536^19^ (GenBank accession number: AJ617685) with a few variations. Given the relatively high error rate of long-read SMRT platforms we amplified 20 specific areas of interest within the gene cluster. Sanger sequencing of the amplified DNA segments identified two misreads in the SMRT sequencing data. Correcting these misreads resulted in a capsule gene cluster which had 99.6% sequence identity with the *E. coli* 536 gene cluster^19^.

The K15 capsular gene cluster from *E. coli* F8316/41 (GenBank accession number: NDK77120.1-NDK77133.1), when screened against the nucleotide collection (nt) database using BLASTN identified 13 different strains of *E. coli* carrying a similar gene cluster in their genome^38^ Of these, genomes of 7 strains were found to be annotated (NCTC11105, FORC_031, FMU073332, 536, 743, MRY15-131 and MRY15-117) and were further analyzed and compared against F8316/41. While all the 7 strains were enterobacterial, MRY15-131 and MRY15-117 were isolated from *Bos taurus*. The capsular gene cluster from all the 8 strains compared earlier belongs to a composite, *pheV*-associated pathogenicity island (PAI). A PAI represents a mosaic-like gene structure with multiple functional and fragmented mobile genetic elements^39^. Fragments of *pheV*-PAI are virulence-associated and highly homologous to chromosomal regions of other entero- and uropathogenic *E. coli*^40^. While DNA sequence and the virulence-associated fragments are a perfect match in *pheV*-PAIs of *E. coli* F8316/41 and *E. coli* NCTC11105 (99.97% identity), they are distinct from the remaining *E. coli* strains listed earlier. Another major distinction between *E. coli* F8316/41, *E. coli* NCTC11105 and the rest of the K15 capsular gene cluster containing E. coli strains is the relative position of *serA*, *pgk* , *pheV* and K15 capsular gene cluster. In *E. coli* F8316/41 and *E. coli* NCTC11105, *serA* and *pgk* genes are located within the *pheV*-PAI with the *pheV* gene placed 44,010 and 58,008 bp upstream to *pgk* and *serA* respectively, while the K15 gene cluster is 32,765 bp downstream to the *serA* gene. On the other hand, the *pheV*-PAI in the remaining six *E. coli* strains (FORC_031, FMU073332, 536, 743, MRY15-131 and MRY15-117) is located upstream to *pgk* which is in turn located upstream to the *serA* gene.

The K15 capsular gene cluster in *E. coli* F8316/41 is flanked by genes *yeeUV* (type IV toxin-antitoxin family)^41^ and *gspM-C* (type II secretion system)^42^. Presence of type II secretion system downstream to capsule gene cluster is the only common feature among all K15 *E. coli* strains discussed here. Pix fimbria-encoding gene cluster and the phosphoglycerate transporter system identified in the *pheV*-PAI of *E. coli* 536^19^ are absent from the *pheV*-PAI of *E. coli* F8316/41. Further in-depth discussion on the composition of *E. coli* F8316/41 *pheV*-PAI is not in the scope of this article. In summation of findings from the genetic analysis section, we identified the K15 capsular gene cluster from *E. coli* F8316/41 as a part of *pheV* associated pathogenicity island. Although the location of PAI in proximity to *serA* gene is consistent with observations from other *E. coli* strains, a different order of arrangement and orientation of genes in PAIs of *E. coli* F8316/41 and NCTC11105 strains point to a divergent evolution from the base *E. coli* genome. As *E. coli* F8316/41 is known to be entero- and uropathogenic, a detailed structural study of PAIs and their genomic elements can help understand the origins of virulence and their role in development of disease.

### Organization of the *E. coli* K15 strain F8316/41 capsule gene cluster

**Figure 6:**
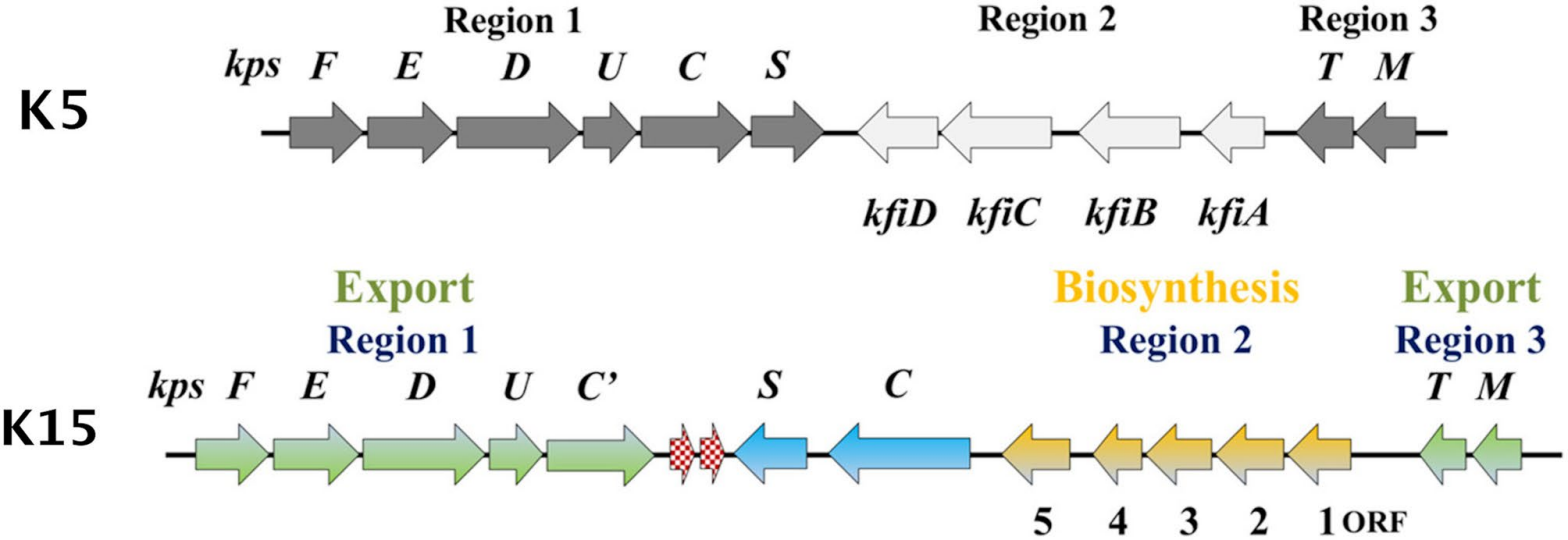
Genetic organization of *E. coli* K15 (F8316-14:O6:K15:H16) capsule gene cluster and its similarity to *E. coli* K5 (Group 2). Regions 1 and 3 (green) are involved in capsule transport across the inner membrane. Region 2 (yellow) is serotype specific, encoding proteins for capsule biosynthesis. ORFs in blue represent the second set of *kpsS* and *kpsC* genes that are of Group 3 origin. Red speckled ORFs are 5′ and 3′ regions of IS630 family transposase. A single point mutation to form a stop codon, resulted in the breakup of a single ORF into 2 fragments.

The arrangement of genes in *E. coli* K15 capsule gene cluster from strain F8316/41 (Fig. 6) shares similarities with other Group 2 capsule gene clusters of the ABC transporter dependent pathway^43^. The gene cluster is made up of three regions, with region 1 containing a total of seven ORFs (*kpsF, -E, -D, -U, -C’* and two unknown putative ORFs). The first five ORFs have a high degree of sequence similarity to other region 1 genes from group 2 capsule forming *E. coli*, except for *kpsC’*, which is a truncated version of *kpsC*. *KpsC’* was reported to be a non-functional gene, inactivated due to truncation.

Regions 2 and 3 are transcribed as a single polycistronic mRNA in the opposite direction to that of region 1, with a JUMPstart sequence located upstream of Region 3^44^. Region 3 codes for KpsM and KpsT, that are homologous to their counterparts of other group 2 capsules. Region 2 is made-up of seven ORFs and flanked by regions 1 and 3. ORFs 1 through 5 are capsule specific genes followed by *kpsC* and *kpsS* that are usually grouped with region 1 genes in group 2 capsule gene clusters. However, these two genes are described to be homologues to *kpsC* and *S* from group 3 capsule forming *E. coli* (K10), and are essential for K15 capsule production^19^. A low G + C content (30.9%) of regions 2 and 3 compared to region 1 (50.9%) of K15 capsular gene cluster was used as evidence to hypothesize origin of these DNA regions from different sources^19^. Functional analysis of capsule specific genes in region 2 was initially performed using a BLASTP search, which identified ORF 1 as a glycosyltransferase and ORF 5 as an α/β hydrolase and the remaining three ORFs as hypothetical *E. coli* proteins without any known functions. As region 2 genes are known to be capsule structure specific, using the K15 PS structure described in the earlier sections, we hypothesized the presence an α-KDO*p* transferase, an α-Glc*p*NAc transferase and an *O*-acetyltransferase among the five ORFs. Although capsules with acetylation and α-Glc*p*NAc units are common, α-KDO*p* is unusual with only two other capsules reported^10,13^.

Enzymes that catalyze capsular *O*-acetylation in bacteria can be classified into one of the following two protein families^45^. The first one is a hexapeptide repeat family, that contains tandem repeats of (LIV)(GAED)X_2_(STAV)X consensus sequence and folds into a left-handed β-helix domain (also termed LβH family)^46^. Members of this family include CssF (both OatW and OatY)^47^ from *N. meningitidis* serogroups W and Y^48^, NeuO from *E. coli* K1^49^ and NeuD from *S. agalactiae*^50^. In addition to the characteristic hexapeptide repeat, enzymes of this family arrange into homotrimers with an active site at each interface. The second family of capsular *O*-acetyltransferases is α/β-hydrolase family. They feature a Ser-Asp-His catalytic triad with a conformationally strained serine located in a conserved nucleophile elbow motif (GX**S**XGG). Previously reported members of this family are CssE (OatC) and CsaC (SacC/MynC) from *N. meningitidis* serogroups C and A respectively^47,51^. Although crystal structure data are lacking for the α/β-hydrolase family, both CssE and CsaC have been thoroughly investigated and reported to catalyze *O*-acetylation of their respective capsular polysaccharide. Upon bioinformatics analysis (BLASTP, pfam, InterPro, etc.)^52–54^, of the five serogroup specific ORFs in region 2 of K15 capsular gene cluster, we identified ORF5 (GenBank accession number: NDK77127.1) to contain an α/β hydrolase fold (pfam-A family: UPF0227 and clan: CL0028, InterPro homologous family: IPR029058, CATH code: 3.40.50.1820) ORF5 encodes a 315 amino acid protein. Sequence alignment with CssE and CsaC (Supplementary Fig. S3) further highlighted a highly conserved catalytic triad composed of Ser-179, Asp-253 and His-281 in addition to the presence of Ser-179 in a conserved nucleophile elbow motif (GG**S**^179^MGG). α/β hydrolases are a versatile family of enzymes that primarily catalyze hydrolytic reactions through a double-displacement mechanism (hydrolases, thioesterases, haloperoxidases, halogenases etc.). There is considerable structural and experimental evidence for α/β hydrolase family catalyzing *O*-acetylation reaction using a Ser-Asp-His catalytic triad (homoserine *O*-acetyltransferase from *H. influenza*)^55,56^. In spite of the sequence similarity between ORF5, OatC and SacC being around 30%, grouping of the three proteins into the same family via bioinformatics analysis, presence of a highly conserved catalytic triad, nucleophile elbow motif, and finally the absence of *O*-acetylation related protein structures in other region 2 ORFs is consistent with ORF5 being the capsular *O*-acetyltransferase.

Identification of specific glycosyl transferases in region 2 for transfer of GlcNAc and KDO was done by searching for capsules from other bacteria, that contained monosaccharide units and glycosidic linkages similar to the K15 PS. In this process, the CPS from *Actinobacillus pleuropneumoniae* serogroup 5a str. J45 (with 6)-α-D-Glc*p*NAc-(1 → 5)-β-KDO*p*-(2 → repeating units) was identified containing a Glc*p*NAc linked to the C5 of KDO*p,* in its α-anomeric conformation^57^. In addition to this CPS structural similarity the capsular gene cluster of *A. pleuropneumoniae* shares a high degree of homology with ABC transporter dependent capsule export genes from *H. influenzae* type b, *N. meningitidis* group B, and *E. coli*^58^. The capsule specific region 2 of *A. pleuropneumoniae* serogroup 5a contains four genes, *cps5A*, *5B*, *5C* and *5D* (sequence AF053723.1)^59^, of which only *cps5A,* (GenBank accession number: AAC26630.1) classified as a putative glycosyltransferase exhibited a 46.2% sequence similarity with ORF1 (GenBank accession number: NDK77131.1) of K15 *E .coli*. This observation along with the absence of similarities between any other region 2 genes of either organisms suggests that ORF1 might encode for an α-Glc*p*NAc transferase. Bioinformatic analysis using primary protein sequence predicted ORF1 to be a member of GT4 family of glycosyltransferases (pfam-A family: Glyco trans 1_4 and clan: CL0113, CDD: cd03801-GT4 PimA-like, CATH code: 3.40.50.2000). As supporting evidence to this hypothesis, a characteristic signature motif, typical to retaining α-glycosyltransferases was observed in the C-terminal end of ORF1 (S211PY**E**GGPACLP**E**ALA225). This conserved secondary structure is an **E**X_7_**E** motif, first reported using hydrophobic cluster analysis^60–62^. The **E**X_7_**E** motif in ORF1 of *E. coli* K15 perfectly aligns with S224SH**E**GGPANIP**E**ALA238 in Cps5A of *A. pleuropneumoniae* (Supplementary Fig. S4). Several other retaining glycosyltransferases belonging to GT4 families carry this conserved motif (for example: *E. coli* lipopolysaccharide α-glucosyltranferase WaaG; *Mycobacterium smegmatis* α-mannosyltransferase, PimA and *Corynebacterium glutamicum* glycosyltransferase, MshA) (Supplementary Fig. S4).

The K15 structure reported previously contains a β-KDO in the repeat unit, thus predicting the presence of β-KDO transferase. Our analysis of the gene cluster did not find evidence of a sequence belonging to the β-KDO transferase family GT-99. The uncommon presence of α-KDO*p* in the capsular polysaccharide made it difficult to use the same approach we employed in identifying α-Glc*p*NAc transferase.

Bioinformatic analysis using CDD, Pfam, dbCAN and InterPro did not generate any hits for ORFs 2,3, and 4. As a result, the second capsule specific glycosyl transferase (α-KDO*p* transferase) was identified using the Phyre^2^ web portal for protein structure modeling and functional analysis. This tool generated hits for ORF4 (GenBank accession number: NDK77128.1) with 100 and 99.5% confidence to Maf (Motility associated factor) glycosyltransferase, from *Magnetospirillum magneticum* AMB-1, and α-2,3/8-sialyltransferase CstII from *Campylobacter jejuni,* respectively. Though the sequence identity was only 19%, models generated with high confidence have been shown to be predictive of structural features and function^63^. Maf glycosyltransferase belongs to a class of bacterial glycosyltransferases involved in transfer of nonulosonic acids like moieties to an acceptor^64^. The Maf central domain has been shown to exhibit similarity to a sialyltransferase for *C. jejuni*^64^. Taking all these findings into account, we predict ORF4 to be an α-KDO*p* transferase with no structural homology to currently documented glycosyltransferases. In summary, we have identified genes in the K15 gene cluster that potentially encode an *O*-acetyltransferase, an *N*-acetylglucosamine transferase, and a KDO transferase consistent with the structure we report. The β-KDO transferases encoded by CPS gene clusters have been reported to belong to the glycosyltransferase family GT99 and have a different fold than KDO transferases associated with LPS biosynthesis^14^. The relationship of the α-KDO transferases of *E. coli* K6, K15, and K16 to these two different families of glycosyltransferases awaits further structural and function analysis.

## Methods

### Growth of the bacteria and preparation of polysaccharide

*Escherichia coli* strain F8316/41 (O6:K15:H16) was obtained from Drs. F. and I. Orskov at Staten Serum Institute, Copenhagen, Denmark. For the isolation of polysaccharide, bacteria were grown on a low molecular weight medium and the polysaccharide was purified by published methods^65^. During isolation, volumes were kept to a minimum to improve yields. Carboxylate reduced polysaccharide was prepared by the carbodiimide-borohydride method of Taylor^66^.

### NMR spectroscopy

20 mg of K15 polysaccharide were dissolved in 0.6 mL of 99.9% D_2_O. The resulting solution was approximately neutral. De-*O*-acetylation of K15 was achieved by increasing the solution pD to ~ 12 by addition of NaOH from a stock solution. NMR experiments were run on a Bruker 700 MHz NMR instrument equipped with a triple gradient TCI cryoprobe. The temperature was set to 50 °C to reduce the line widths. The internal reference standard was ~ 0.1 (wt %) DSS-d_6_. NMR spectra were obtained using Bruker’s TopSpin 3.5 software (www.bruker.com), analysis was performed with both Topspin and Mnova 11 (www.mestrelab.com). A spin-lock time of 60 ms was used in the HSQC-TOCSY experiment. For the evolution of long-range couplings in the ^1^H,^13^C HMBC experiment a *J*_H,C_ coupling constant of 6 Hz was used.

### Other analytical methods

Sugar components were detected by paper chromatography, paper electrophoresis, gas chromatography and automated sugar analysis. Thin layer chromatography was performed on TLC-cellulose plates in butanol-pyridine-water, 6/4/3, v/v/v^67^. Hexosamine was released by hydrolysis in 4 N HCl. KDO was quantified in hydrolysates by the thiobarbituric acid assay. Alditol acetates were prepared and analyzed as described previously^65^.

### Glycosyl composition

Glycosyl composition analysis was performed by combined gas chromatography/mass spectrometry (GC/MS) of the per-*O*-trimethylsilyl (TMS) derivatives of the monosaccharide methyl glycosides produced from the sample by acidic methanolysis as described previously by Santander et al.^68^. Briefly, the samples (230 and 240 μg) were heated with methanolic HCl in a sealed screw-top glass test tube for 17 h at 80 °C. After cooling and removal of the solvent under a stream of nitrogen, the samples were treated with a mixture of methanol, pyridine, and acetic anhydride for 30 min to re–*N*-acetylate the hexosamines. The solvents were evaporated, and the samples were derivatized with Tri-Sil (Pierce) at 80 °C for 30 min. GC/MS analysis of the TMS methyl glycosides was performed on an Agilent 7890A GC interfaced to a 5975C MSD, using a Supelco Equity-1 fused silica capillary column (30 m 0.25 mm ID).

### Degradation procedures

Oligosaccharides were prepared by mild acid hydrolysis in 1% acetic acid at 100 °C for 1 h. The resulting disaccharide was purified by gel filtration on Bio-Gel P-2 in 0.1 M ammonium acetate and subsequent paper electrophoresis in pyridine acetate, pH 5.4. K15 was oxidized with excess sodium metaperiodate for 40 h at 4 °C and desalted after quenching. Oxidized polysaccharide was reduced with sodium borohydride at pH 7.0 on a pH stat. Beta elimination of periodate oxidized polysaccharide was performed as follows. Oxidized polysaccharide was dialyzed, lyophilized, and then treated with 0.1 N sodium hydroxide at 37 °C for 2 h. The reaction mixture was applied to a TLC cellulose plate in butanol-pyridine-water, 6/4/3, v/v/v.

Hexosamine released by acid hydrolysis was degraded to a pentose by ninhydrin degradation in pyridine as described previously^20^. The products were identified by automated sugar analysis.

### Glycosyl linkage analysis

For glycosyl linkage analysis, the samples were permethylated, reduced, hydrolyzed under mild conditions, reduced, hydrolyzed again and acetylated; and the resultant partially methylated alditol acetates (PMAAs) analyzed by gas chromatography-mass spectrometry (GC–MS). The procedure is a slight modification of the one described by Willis et al.^69^.

About 500 μg of the samples were used for linkage analysis. The samples were suspended in 200 μl of dimethyl sulfoxide and left to stir for 1 day. Permethylation of the sample was affected by two rounds of treatment with sodium hydroxide (15 min) and methyl iodide (45 min). The permethylated sample carboxylic acids were reduced by adding 200 μl of a 5 mg/ml solution of LiBD_4_ in 90% THF and reacting overnight at room temperature, followed by 1 h at 100 °C. The samples were then hydrolyzed using 0.1 M TFA (0.5 h in sealed tube at 100 °C), reduced with NaBD_4_, hydrolyzed again using 2 M TFA (2 h in sealed tube at 100 °C) and acetylated using acetic anhydride/TFA. The resulting PMAAs were analyzed on an Agilent 7890A GC interfaced to a 5975C MSD (mass selective detector, electron impact ionization mode); separation was performed on a 30 m Supelco SP-2331 bonded phase fused silica capillary column.

### Whole genome sequencing and capsule cluster analysis

The chromosomal DNA isolated from *E. coli* str. F8316/41 was sequenced using single-molecule real-time (SMRT) sequencing technology^34^. The raw sequencing data was processed using Canu v. 1.5^70^. Canu is a long read single-molecule sequence de novo assembly tool for PacBio and/or Nanopore reads. This process resulted in five contiguous DNA segments with base lengths as follows: 2,558,639, 2,393,415, 99,428, 88,757, and 29,847 bases (GenBank accession numbers: JAADZB010000001 – JAADZB010000005). The second contiguous DNA segment with 2,393,415 bases was amplified in 20 different regions using forward and reverse primers (see Supplementary Information) to perform Sanger sequencing. The contiguous DNA sequences were annotated using NCBI’s Prokaryotic Genome Annotation Pipeline (PGAP) and submitted to GenBank^71^. The K15 capsular gene cluster and the *pheV*-PAI genetic region were analyzed and compared with genomes of other E. coli K15 strains using BLASTN^38^ and BLASTP^53^. The ORFs in the K15 gene cluster were generated using ORFfinder (https://www.ncbi.nlm.nih.gov/orffinder/). A wide array of bioinformatic resources like the pfam database^52^, CDD search^72^, InterPro classification of protein families^54^, CATH/Gene3D v4.2^73^, Phyre^2^ protein fold recognition server^74^ and dbCAN meta server^75^ for automated CAZyme annotation were used for identification and functional characterization of capsule specific region 2 genes.

## Supplementary information

Supplementary Information.
