# Supplementary material for: Chemical structure and genetic organization of the E. coli O6:K15 capsular polysaccharide
Source: Sci Rep. 2020 Jul 28;10:12608. doi: 10.1038/s41598-020-69476-z (PMC7387560; doi:10.1038/s41598-020-69476-z)
Supplement: Supplementary file 1 — Supplementary Information. [file 41598_2020_69476_MOESM1_ESM.pdf]

Supplementary Information

Chemical Structure and Genetic Organization of the *E. coli* O6:K15 Capsular Polysaccharide

Hugo F. Azurmendi[1], Vamsee Veeramachineni[1], Stephen Freese[2], Flora Lichaa[1], Darón I. Freedberg[1] and Willie F. Vann[1*]

[1]Laboratory of Bacterial Polysaccharides, Food and Drug Administration, Silver Spring, MD,
[2]Affinivax, 650 East Kendall St, Cambridge, MA 02138

Content:

- **Primers used to amplify K15 capsular gene cluster in E. coli F8316/41 Contig2**
- **Table S1.** Survey of ^{1}H chemical shifts (δH, ppm) for α- and β-KDO H3e,a.
- **Table S2.** Genes and genomes used to the genetic analysis.
- **Figure S1.** Overlay of K15 HSQC and HSQC-TOCSY experiments.
- **Figure S2.** NMR assignments of native K15 polysaccharide and quantitation of *O*-acetylation by 1D ^{13}C NMR.
- **Figure S3.** Sequence alignments of ORF5 with α/β hydrolase family of capsular *O*-acetyltransferases.
- **Figure S4.** Sequence alignments of ORF1 with similar Glycosyltransferases.

Primers used to amplify K15 capsular gene cluster in E. coli F8316/41 Contig2:

K15 CPS Forward Primer 1: 5'-CGCGAGAGTTTAGTCTCTCATT-3'
K15 CPS Reverse Primer 1: 5'-GCTCGGTGTTGAACTCTACTT-3'

K15 CPS Forward Primer 2: 5'-GCCAGCAAAGCGGAATTG-3'
K15 CPS Reverse Primer 2: 5'-ACCAGAGTGTTCAACTGACTATT-3'

K15 CPS Forward Primer 3: 5'-GAAAGCAGCATTCCCGTAAC-3'
K15 CPS Reverse Primer 3: 5'-GAGCCGGGATGACAATAACA-3'

K15 CPS Forward Primer 4: 5'-CGAGCCAAGCACGGTAAA-3'
K15 CPS Reverse Primer 4: 5'-TCTCGGCAATCAAACGTACTC-3'

K15 CPS Forward Primer 5: 5'-TGCAAACGGCAACAAACC-3'
K15 CPS Reverse Primer 5: 5'-CTGGTTCATCACCCACAGTT-3'

K15 CPS Forward Primer 6: 5'-AAACGGCGGCTCAGACA-3'
K15 CPS Reverse Primer 6: 5'-TCATGACAAATCCCATGACTACA-3'

K15 CPS Forward Primer 7: 5'-GACTCACTGACATCCCGAATTA-3'
K15 CPS Reverse Primer 7: 5'-CTGAAACACTATCGCTACCCTTAT-3'

K15 CPS Forward Primer 8: 5'-ACCGCTCCCTTTGTTCATAC-3'
K15 CPS Reverse Primer 8: 5'-GGAAGCCCATTTATCCGTAGTA-3'

K15 CPS Forward Primer 9: 5'-CCACTCAGCGAAGTCATTGTA-3'
K15 CPS Reverse Primer 9: 5'-CGTTTCTTCTCAAGTTGGGTTTG-3'

K15 CPS Forward Primer 10: 5'-CCAACCAGCATAGAATGGCA-3'
K15 CPS Reverse Primer 10: 5'-TCACTATCTTTATTGAGCCCTGTT-3'

K15 CPS Forward Primer 11: 5'-GATCAGAGACACGGCTTTCC-3'
K15 CPS Reverse Primer 11: 5'-TTGAAGCAGCCTCTCTTCATAA-3'

K15 CPS Forward Primer 12: 5'-TGATTGAAACCATGAAGGATCT-3'
K15 CPS Reverse Primer 12: 5'-GACCTTATTTGCCGTACTCAGA-3'

K15 CPS Forward Primer 13: 5'-CCTGATGTTCACAAATAGGCATAC-3'
K15 CPS Reverse Primer 13: 5'-GCTGCTGGGAATGTTGTTATT-3'

K15 CPS Forward Primer 14: 5'-TCTTCCTCCCACTCGGTTAT-3'
K15 CPS Reverse Primer 14: 5'-GGTCGAAAGGTATTGATGTATGC-3'

K15 CPS Forward Primer 15: 5'-TCCCTGAATCCAATAAAGTCAT-3'
K15 CPS Reverse Primer 15: 5'-ATTTGAACAGTGCTCATTTCTGG-3'

K15 CPS Forward Primer 16: 5'-GCCAGCAATTACCAACCAAATA-3'
K15 CPS Reverse Primer 16: 5'-TCACCAGATAGTTGCGATTACAT-3'

K15 CPS Forward Primer 17: 5'-AGCTAGCAGAACTGCTGAAA-3'
K15 CPS Reverse Primer 17: 5'-TGATGGCAATTCGAGATATGTA-3'

K15 CPS Forward Primer 18: 5'-GGATAGAACCTTATATCGAGCACTT-3'
K15 CPS Reverse Primer 18: 5'-GCAATGGAGCAGGAAAGTCTA-3'

K15 CPS Forward Primer 19: 5'-GCTATAATTGTATCTATAGGCCTTACTG-3'
K15 CPS Reverse Primer 19: 5'-GAGGGATAATGCTGGCTGTAA-3'

K15 CPS Forward Primer 20: 5'-GAACGCACGCGATTGATTAC-3'
K15 CPS Reverse Primer 20: 5'-AACGCCAGCACAGGTATC-3'

Table S1. Representative survey of ^{1}H chemical shifts (δH, ppm) for α- and β-KDO H3e,a. The following rules are observed: (i) for αKDO δH3e < 2.3 ppm and Δδ < 0.4 ppm; [1] for β-KDO δH3e > 2.3 ppm and Δδ > 0.4 ppm.

α/β	Compound and reference	δH3e	δH3a	Δδ
αKDO (αK)	OH-**αK** [2]	2.06	1.79	0.27
	CH_3-**αK** [3]	2.06	1.79	0.27
	K16 (…**-5αK2-**…) [3]	2.17	1.82	0.35
	**K15 (…-5αK2-…)** (this work)	**2.098**	**1.887**	**0.21**
	**αK2**-4αK2-allyl,[4]	2.24	1.87	0.37
	αK2-**4αK2**-allyl,[4]	2.10	2.02	0.08
	**αK2**-8αK2-allyl,[5]	2.03	1.79	0.37
	αK2-**8αK2**-allyl,[5]	2.05	1.79	0.34
βKDO (βK)	CH_3-**βK** [3]	2.38	1.74	0.64
	K12 (…**-5βK2-**…) [3]	2.45	1.99	0.46
	K19 (…**-5βK2-**…) [3]	2.56	1.90	0.66
	**βK2**-4βK2-…[6]	2.45	1.86	0.69
	βK2-**4βK2**-…[6]	2.35	1.81	0.54
	**-4βK2**-7βK2-…[6]	2.32	1.81	0.51
	-4βK2-**7βK2-**…[6]	2.46	1.87	0.59

1. Blattner, F.R. et al. Complete genome sequence of Escherichia coli K-12. *Science (Washington, D. C.)* **277**, 1453-1462 (1997).
2. Birnbaum, G.I., Roy, R., Brisson, J.-R. & Jennings, H.J. Conformations of Ammonium 3-Deoxy-D-manno-2-octulosonate (KDO) and Methyl α- and β-Ketopyranosides of KDO: X-Ray Structure and 1H NMR Analyses. *Journal of Carbohydrate Chemistry* **6**, 17-39 (1987).
3. Lenter, M., Jann, B. & Jann, K. Structure of the K16 antigen from Escherichia coli O7:K16:H-, a Kdo-containing capsular polysaccharide. *Carbohydr Res* **197**, 197-204 (1990).
4. Haselhorst, T. et al. NMR experiments reveal distinct antibody-bound conformations of a synthetic disaccharide representing a general structural element of bacterial lipopolysaccharide epitopes. *Biochemistry* **38**, 6449-6459 (1999).
5. Sokolowski, T. et al. Conformational analysis of a Chlamydia-specific disaccharide alpha-Kdo-(2-->8)-alpha-Kdo-(2-->O)-allyl in aqueous solution and bound to a monoclonal antibody: observation of intermolecular transfer NOEs. *J Biomol NMR* **12**, 123-133 (1998).
6. Ovchinnikova, O.G. et al. Biochemical Characterization of Bifunctional 3-Deoxy-beta-d-manno-oct-2-ulosonic Acid (beta-Kdo) Transferase KpsC from Escherichia coli Involved in Capsule Biosynthesis. *J Biol Chem* **291**, 21519-21530 (2016).

Table S2: Accession numbers of genes and genomes from various *E. coli* strains used in the genetic analysis section.

#	Gene/Protein Name	Organism & Strain	Accession number	Base pair gene location on genome
1	*E. coli* F8316/41 Contig1	*E. coli* F8316/41	JAADZB010000001	NA
2	*E. coli* F8316/41 Contig2	*E. coli* F8316/41	JAADZB010000002	NA
3	*E. coli* F8316/41 Contig3	*E. coli* F8316/41	JAADZB010000003	NA
4	*E. coli* F8316/41 Contig4	*E. coli* F8316/41	JAADZB010000004	NA
5	*E. coli* F8316/41 Contig5	*E. coli* F8316/41	JAADZB010000005	NA
6	*pgk*	*E. coli* F8316/41	NDK77077.1	NA
7	*pheV*	*E. coli* F8316/41	NA	1751755-1751830
8	*serA*	*E. coli* F8316/41	NDK77088.1	NA
9	*pheV'*	*E. coli* F8316/41	NA	1842624-1842644
10	Capsular gene cluster	*E. coli* F8316/41	NDK77120.1-NDK77133.1	NA
11	*gspM-C* (type II secretion system)	*E. coli* F8316/41	NDK77134.1-NDK77144.1	NA
12	*E. coli* 536 *pheV*-PAI	*E. coli* 536	AJ617685.1	NA
13	*E. coli* 536 Whole genome	*E. coli* 536	CP000247.1	NA
14	*E. coli* NCTC11105 Whole genome	*E. coli* NCTC11105	LR134157.1	NA
15	*E. coli* FORC_031 Whole genome	*E. coli* FORC_031	CP013190.1	NA
16	*E. coli* FMU073332 Whole genome	*E. coli* FMU073332	CP017844.1	NA
17	*E. coli* 743 Whole genome	*E. coli* 743	CP015069.1	NA
18	*E. coli* MRY15-131 Whole genome	*E. coli* MRY15-131	AP017620.1	NA
19	*E. coli* MRY15-117 Whole genome	*E. coli* MRY15-117	AP017617.1	NA

Figure S1 NMR assignments of the K15 CPS at pD ~12 and T = 50°C. HSQC (red) overlaid with HSQC-TOCSY spectrum acquired with a 60 ms mixing time (blue), showing the ^{1}H-^{1}H spin coupling at different ^{13}C chemical shifts. Horizontal dashed lines highlight examples of ^{1}H-^{1}H spin systems: at GlcNAc C1 (98.55 ppm, indicated by G1), GlcNAc C2 (53.97 ppm, indicated by G2) and KDO C3 (34.75 ppm, indicated by K3) ^{13}C chemical shifts. The spin sytem for G1 is enlarged on top to visualize the G(H5,C1) correlation more clearly.

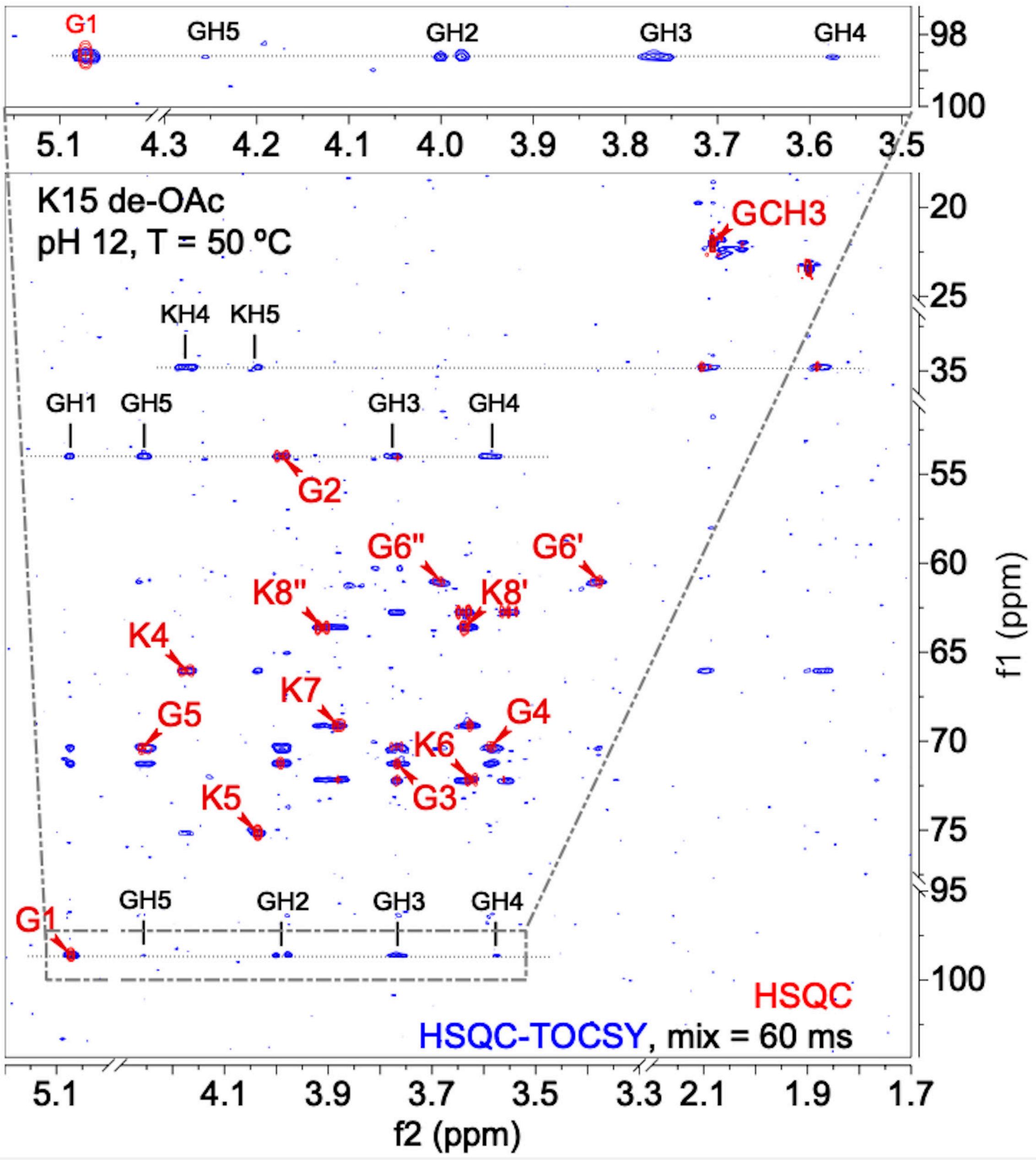

Figure S2 A) Overlay of HSQC experiments of the native (blue) and de-*O*-acetylated (red, with black labels) K15 PS. Only two reassigned peaks (with the largest changes in chemical shifts) are labeled in blue for K15(G3+OAc), indicated as G3A and G4A. The dashed arrow indicates the change in the G3 resonance due to *O*-acetylation. B) Quantitation of *O*-acetylation of K15 by integration of signals on an inverse-gated ^{13}C experiment.

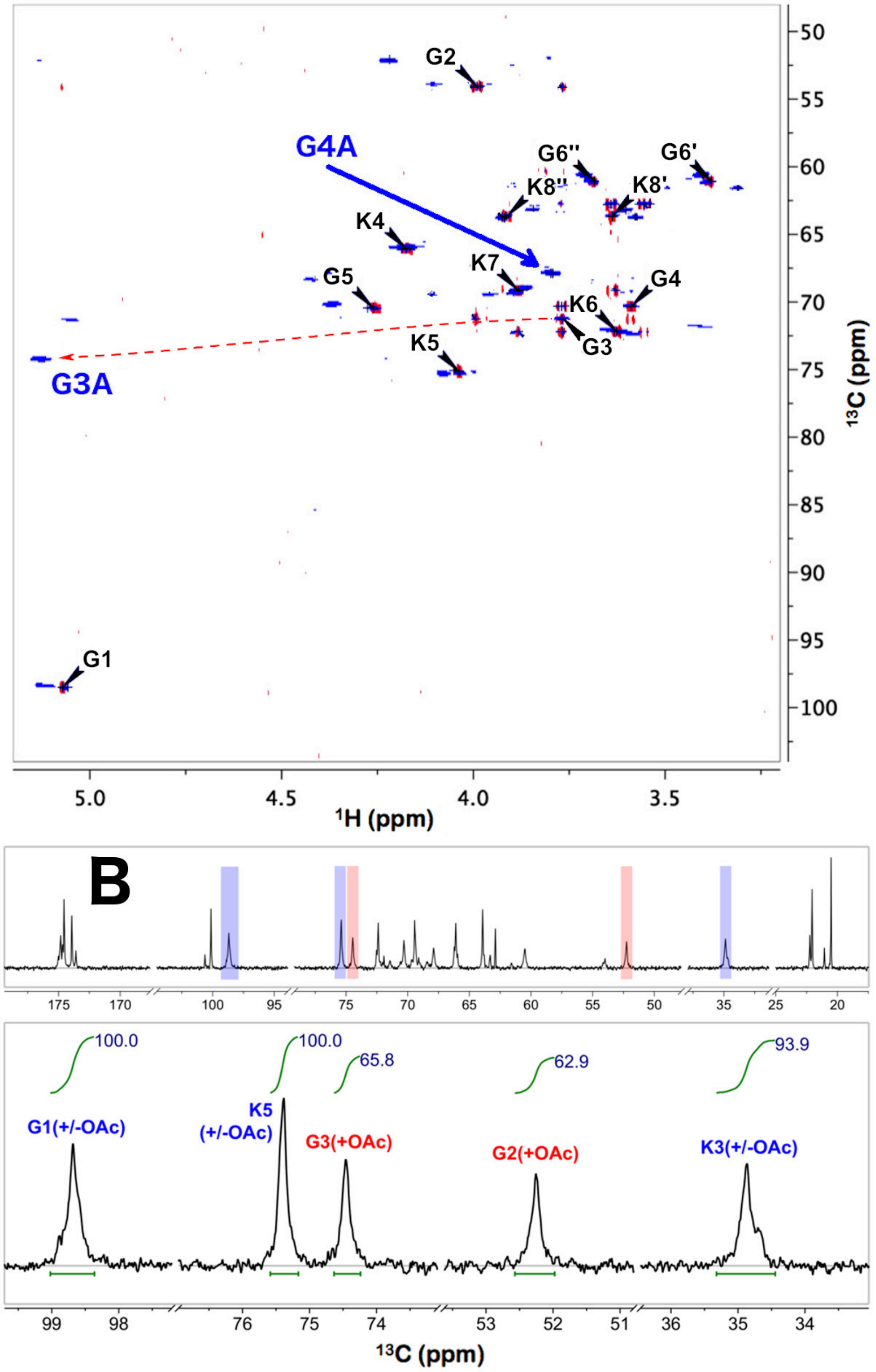

Figure S3. Sequence alignment of ORF5 with α/β hydrolase family of capsular *O*-acetyltransferases: Multiple sequence alignment between ORF5, CsaC (SacC/MynC) of *N. meningitidis* serogroup A and CssE (OatC) of *N. meningitidis* serogroup C was performed using Clustal Omega. Completely conserved aminoacids are labeled in red. Aminoacids labeled in green and purple indicate strong and weak properties similarities, respectively. The orange box emphasizes highly conserved nucleophile elbow motifs. Conserved residues of Ser-Asp-His catalytic triad are indicated with blue triangles.

```
ORF5      ------------------------------------------------------------ 0
CsaC      ------------------------------------------------------------ 0
CssE      MSINTFETFIYELYQSTNFAEIARITGFSNHKALNLETIRMMAISCFKTKNTTKCIELSE 60

ORF5      -------------------------------------------------------MKFMN 5
CsaC      ------------------------------------------------------------ 0
CssE      YFNEKSDIKDFFIFEILAECYFLQNDLVKSLENYEKALLLNPKLISARFKHMCLKYRLFN 120

ORF5      T----------------QEI--------INFTAENYMNDNG--------SPELNYQDYLK 33
CsaC      ------------------------------------------------------------ 0
CssE      ELDSTTFSKYEIESQQKKKISNLRIIAYIQLKEKKYFKAYNNLKLLIELNKSPFYPDYLS 180

ORF5      IYRSWEVYSPSLDKIRNRLPLKDSVVKLVYKKIYSKMPFDREYILKKYEHTYKYIDYLLY 93
CsaC      --------------------------MLSNLKTGNNILGLPEFELNGCR-------FLYK 27
CssE      IIHAIENLEYSKQSQNTKKE------LTEYIKISKAIALKSEFVCNGSN-------NLFV 227
                                         *  . :    *:  :  .        *

ORF5      PSINAERLVVLLSGYSQRKTYNRYSWFWDDTEKWDGSTAY-LF------LNDTENTWYCG 146
CsaC      K--GIEKTIITFSAFPPKDIAQKYNYIKDFLS---SNYTFLAFLDTKYPEDDARGTYYIT 82
CssE      ----------TLSP-ATGFVLKKYNYPADKL----------CFI-------DNTNTYYT- 258
                     :*        ::*.:  *             *        *  .*:*
                                          179
ORF5      HENQKINVYKE-VIFLISNRYKLDNRKIYIVGGSMGGYAALRLGLELNLGG--V----IS 199
CsaC      NELD--NGYLQTIHCIIQLLSNTNQEDTYLLGSSKGGVGALLLGLT--YNYPNIIIN--- 135
CssE      ------FAYELIAEHIISLVKKYKYENISIIGSSKGGTATIILLNLLQTALPNTTICAVS 312
                  *      :*.   : . ..  ::*.* ** .:: *

ORF5      INPQTSLEAASLH---KDPSW-------FQSINKCGENFIPVAE------IIRRNEPTKI 243
CsaC      -APQAKLADYIKTRSKTILSYMLGTSKRFQDINYDYINDFLLSKIKTCDSSLKWNIH--- 191
CssE      CSPQIQIFPFNK--NLTIPSY-----QKFA--EYFSYNSILESKCAQAQKLINFDILYRN 363
            ** .:         .  *:       *   :    * :  ::       :. :
                  253                             281
ORF5      YLECGDYLADSFDLESISQAIVEKG----GLFILNHHSSDKHV----TSSPNKEQLDMLI 295
CsaC      -ITCG--KDDSYHLNELE--ILKNEFNIKAITIKTKLISGGHDN---------EAIAHYR 237
CssE      KLTIF--YGDKFKMDAQEVSTIRPINN---TTII-PLNYSGHGSLIPLTIPENKSFDELK 417
           :       *.:.::  .   :.         *      . *           : :

ORF5      SFFSDDTISNL--AAENNVLSS---------------------                  315
CsaC      EY-----------------FKTIIQNI----------------                  247
CssE      QKYSKLEIDTDFQALGGNNLSSIVDEIFEIYSNPDMRLHKFLC                  460
          .                  :.:
```

Figure S4. Sequence alignment of ORF1 with similar Glycosyltransferases: Multiple sequence alignment between ORF1, Cps5A of *A. pleuropneumoniae, E. coli* lipopolysaccharide α-glucosyltranferase (WaaG), *M. smegmatis* α-mannosyltransferase (PimA) and *C. glutamicum* glycosyltransferase (MshA) was performed using Clustal Omega. Completely conserved aminoacids were labeled in red. Aminoacids labeled in green and purple indicate strongly and weakly similar properties respectively. Orange box was used to highlight conserved primary sequence EX_7E that is common among these α-glycosyl transferases (identified using hydrophobic cluster analysis).

```
ORF1    -------------MTVLINHVMST---------------------------NVKSSIFRDF 21
Cps5A   ----------SIMTRPIINHVMSR--------------------------DIQSGIFSSI 26
WaaG    ----------KVPVKSHTNHG------------------------------RNAEYFAWV 72
PimA    DYVVSGGKAVPIPYNGSVA-------------RLRFGPA------------THRKVKKWI 83
MshA    ENL-RVINIAAGPYEGLSKEELPTQLAAFTGGMLSFTRREKVTYDLIHSHYWLSGQVGWL 119
                                                                   .
ORF1    IFRIKENSLSCFQHTVSIEPKSYADIYHFHRPQKCDIEQIPEKSLVTLHFDPFDFRQHSS 81
Cps5A   LEYFTDFGSNEFQHIVSVSPIPEAKVYHYHRPHLEEK--LLPNSVCTVHHDLNDPDPWHA 84
WaaG    QKHL---REHPVDKVVGFNKMPGLDVYYAADVCYAEKVAQEKG---FFYRLTSRYRHYAA 126
PimA    AEGD---FDVLHIHEP---NAPSLSML-----ALQAAEGPIVA---TFHTSTTKSLTLSV 129
MshA    LRDL---WRIPLIHTA---HTLAA----------------VKN---SYRDDSDTP--ES- 151
                     :
ORF1    M---EELFQKLLLFKKVVFLN---QNSF-----EQCSFLGEKRVLIPHGYDERLQLRQQK 130
Cps5A   K---YRFIPRYMEAGAIICLNYTQKEIL-----ISQGLPEHKLFVIPHGYNQKVLFPKKI 136
WaaG    FE--RATFE---QGKPTQLLMLTDKQIADFQKHYQTEAE--RFHILPPGIYPDRKYSQQP 179
PimA    FQGILRPYHEKIIGRI---------AVSDLARRWQMEALGSDAVEIPNGVDVAS-FA--- 176
MshA    --EARRICEQQLVDNADVLAVNTQEEMQDLMHHYDADPD--RISVVSPGADVEL-YSPGN 206
                                          .          :  *

ORF1    KP---HFTFNVA--------VISRYYSDGRKGDAYLFDLLAGLP----NDI-RLYLVGNC 174
Cps5A   KEISSTDKITLG--------IASRRYGRRVKGDAYLFELAKRLN----PDHFKFIFVGKD 184
WaaG    ANSREIFRKKNGITEQQYLLLQVGS-DFTRKGVDRSIEALASLPDSLRHNTLLYVVGQ-- 236
PimA    -----DAPLLDGYPREGRTVLFLGRYDEPRKGMAVLLAALPKLVARFPDVEILIVGRGDE 231
MshA    DRATERSRRELGIPLHTKVVAFVGRL-QPFKGPQVLIKAVAALFDRDPDRNLRVIICGGP 265
                   .                  **    :     *
                                                           EXXXXXXXE
ORF1    W-------PKR----LEKRSKQIYVIHPTSYYDIIRIYSAIDMVLITSP-YEGGPACLPE 222
Cps5A   R-------QYSAL-EMQDLGFEAQVYERLPYRMFQSFYNNIDVLLMCSS-HEGGPANIPE 235
WaaG    ----DKPRKFEALAEKRGVRSNVHFFSGRNDV--SELMAAADLLLHPAY-QEAAGIVLLE 289
PimA    D-------ELREQAG--DLAGHLRFLGQVDDATKASAMRSADVYCAPHLGGESFGIVLVE 282
MshA    SGPNATPDTYRHMAEELGVEKRIRFLDPRPPSELVAVYRAADIVAVPSF-NESFGLVAME 324
                             .  .                *:        *.      *

ORF1    ALAAGCLVFSSRSGMAEDLLDTR---FLLDYNLTSD---INKILMA---K-SLFKNEIE- 271
Cps5A   ALATGTPIFSSNIGIPKDVVINYKNGLILTLDPDIDAEQINFICLE---KPNIFENILDF 292
WaaG    AITAGLPVLTTAVCGYAHYIVDANCGEAIAEPFRQ--ETLNEILRKALTQSSLRQAWAEN 347
PimA    AMAAGTAVVASDLDAFRRVLADGDAGRLVPVDD-A--EGMAAALIEILEDDQLRAGYVAR 339
MshA    AQASGTPVIAARVGGLPIAVAEGETGLLVDGHS-P--HAWADALATLLDDDETRIRMGED 381
        * ::*  :.::        :        :                    . .

ORF1    --NYKAHNLITWETVSHRYNCLYQSML----------------------            296
Cps5A   SLKQSPSLAISWEKCIQQNILVYKKIIKG--------------------            321
WaaG    --ARHYADTQDLY----SLPEKAADIITG----GLDG------------            374
PimA    --ASERVHRYDWSVVSAQIMRVYETVSGAGIKVQVSGAANRDETAGESV            386
MshA    --AVEHARTFSWAATAAQLSSLYNDAIANE---NVDGETHHG-------            418
```
